# Ensuring safety for children with trachomatous trichiasis: experiences from South Sudan

**Published:** 2024-02-09

**Authors:** Angelia Sanders, Maurice Abony, Brendan Callahan, Albino Nyibong, Yak Yak Bol

**Affiliations:** 1 Associate Director: Trachoma Control Program, The Carter Center, Atlanta, USA.; 2 NTD Programme Manager Africa East and South: CBM International, Nairobi, Kenya.; 3 Senior Program Manager: HCP Cureblindness, Burlington, Vermont, USA.; 4 Director of Eye Services: Ministry of Health, Juba, Republic of South Sudan.; 5 National Coordinator for PC-NTDs: Ministry of Health, Juba, Republic of South Sudan.

**Hospitals and trained personnel are needed to ensure that children receive appropriate surgery for trachomatous trichiasis; investment in integrated care can make this possible.**

**Figure:**
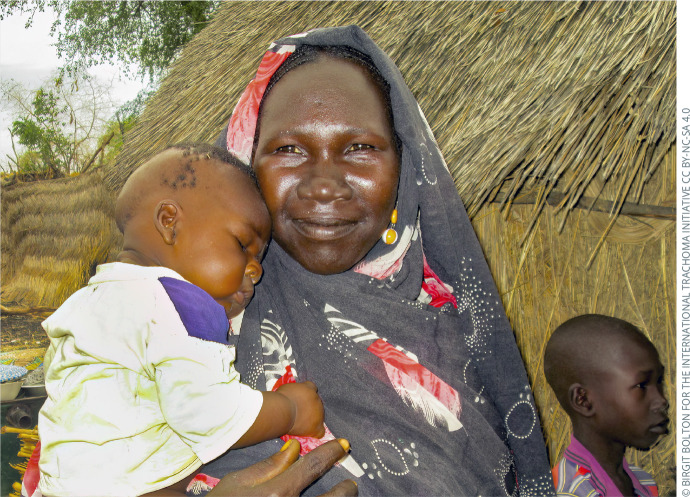
Investment in regional eye health centres will improve eye care services for children. south sudan

Trachoma, the world's leading infectious cause of blindness, is caused by the bacterium *Chlamydia trachomatis*. Trachoma manifests in young children as a chronic inflammation of the eyelid. Repeated infection can result in scarring of the eyelid and the development of trachomatous trichiasis (TT), a condition whereby the eyelid turns inward, causing eyelashes to painfully scratch the eye's surface. If left untreated, TT can result in irreversible vision impairment and blindness. 

TT typically develops in adulthood, after years of repeated trachoma infection throughout childhood and adolescence. However, in South Sudan, during a ten-day eye care surgical outreach campaign conducted in May 2023 in the trachoma hyper-endemic county of Uror, Jonglei state, 85 children under the age of 12 were identified with TT. This presented several challenges to the national trachoma programme, requiring further reflection to ensure TT is managed safely and effectively in children.

A major challenge for the programme is to ensure the correct diagnosis and documentation of TT in children. In many cases, children are assumed to have distichiasis (extra eyelashes that face inward from an otherwise normal eyelid), which is more common than TT in children. This differential diagnosis can result in an underestimate of the TT burden in children and prevent or delay children receiving the appropriate treatment.

The surgical management of TT in children differs from that for adults. In general, adults receive day surgery, using local anaesthesia. Typically, for adults, these operations are performed in the community, during outreach campaigns. Follow-up assessments are ideally conducted on the day after surgery, and again at 7–14 days and 3–6 months after surgery.^1^ Children, on the other hand, should be managed in a hospital and not in a community setting, as they usually require general anaesthesia to undergo TT surgery. Additionally, children are usually kept in a health care setting at least overnight and sometimes longer. These arrangements are needed to minimise the distress that could be caused to the child and to ensure they remain still during surgery to reduce the risk of adverse events.

Given the limited eye care facilities in South Sudan, managing paediatric TT often requires transportation so that children (and their caregivers) can be treated in health facilities with appropriate equipment and trained health personnel who can perform the surgery under general anaesthesia. This can require travel to major cities, such as Juba, or neighbouring countries, such as Uganda. This is expensive and logistically difficult for national trachoma programmes. 

To improve the provision of eye health services, South Sudan's Ministry of Health is providing integrated eye health packages in Uror county. This includes trachoma interventions alongside other eye health services, such as cataract surgery. This approach has improved the availability of human resources for eye health, reduced costs, and improved the patient experience by ensuring people with eye health issues are not turned away for not having the specific eye health issue being treated by a particular programme. Despite this approach, there is frequently a lack of trained personnel who can provide these services to children.

Going forward, there is a need to build up the presence and capacity of regional eye health centres and provide them with the resources to conduct paediatric ophthalmic surgery. This includes increasing access to anaesthetists who are sufficiently trained and equipped to perform general anaesthesia on children. Given that the actual TT surgery is very similar in adults and children, the current cadre of TT surgeons can typically also perform paediatric TT surgery; however, national programmes would benefit from additional training of eye care workers to ensure that children with TT are correctly diagnosed and appropriately managed.

Investing directly in health systems will enable specialised eye health workers and sub-specialists, including anaesthetists, to serve the most remote and hard-to-reach communities. This will have a significant long-term benefit for communities at risk of trachoma by improving the diagnosis of TT in children and reducing the need to transfer patients to Juba or neighbouring countries to be treated by specialised health workers. 

The International Coalition for Trachoma Control preferred practice titled Supportive Supervision for TT Programs, published in 2016, recommends that programmes do not conduct surgery on children in outreach settings.^2^ The experience in South Sudan highlights that TT is present in children and underscores the importance of established protocols to ensure that all children are treated safely and effectively in appropriate health facilities, such as hospitals equipped and staffed to deliver general anaesthesia for children.
